# Generalized Uncoupled Bone Remodeling Associated With Delayed Healing of Fatigue Fractures

**DOI:** 10.1002/jbm4.10598

**Published:** 2022-01-19

**Authors:** Xiaoyu Tong, Mikael J Turunen, Inari S Burton, Heikki Kröger

**Affiliations:** ^1^ Kuopio Musculoskeletal Research Unit (KMRU), Clinical Research Centre, Institute of Clinical Medicine, University of Eastern Finland Kuopio Finland; ^2^ Department of Applied Physics University of Eastern Finland Kuopio Finland; ^3^ Department of Orthopaedics, Traumatology, and Hand Surgery Kuopio University Hospital Kuopio Finland

**Keywords:** STRESS FRACTURE, FATIGUE FRACTURE, PERITRABECULAR FIBROSIS, HISTOMORPHOMETRY, FTIRI, BONE REMODELING

## Abstract

Fatigue fractures in bones are common injuries with load‐bearing activities, during which the remodeling aimed at removing microdamage has been suggested to play a role in increasing related fracture risk. Much attention has been given to the uncoupling between osteoclastic bone resorption and osteoblastic osteogenesis in fatigue fracture cases; however, the underlying pathophysiologic mechanisms of impaired fracture healing are yet unknown. Here we report multiple fatigue fractures in a physically active woman receiving contraceptive pills for years. Her fracture healing was remarkably slow, although she has been otherwise healthy. The patient underwent bone biopsy of the iliac crest that showed remarkable peritrabecular fibrosis with increased osteoclastic bone resorption combined with relatively low bone formation. Analysis of bone biochemical composition revealed a more complex picture: First, notable declines in bone mineral content–based parameters indicating abnormal mineralization were evident in both cancellous and cortical bone. Second, there was elevation in mineral crystal size, perfection, and collagen maturity in her bone tissues from different anatomical sites. To our knowledge, this is the first report showing generalized uncoupling in bone remodeling, increased peritrabecular fibrosis, and bone compositional changes associated with delayed healing of fatigue fractures. These results may explain delayed healing of fatigue and stress fractures. © 2021 The Authors. *JBMR Plus* published by Wiley Periodicals LLC on behalf of American Society for Bone and Mineral Research.

## Graphical abstract

Methodological presentation of the iliac crest biopsy processing.

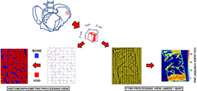

## 1 Introduction

Stress fracture refers to the repetitive strains on bone, leading to material fatigue, microarchitectural discontinuities, and fracture thereafter.^(^ ^1^ ^)^ Its proper definition and pathophysiology could be described as two contrary processes with a similar result: an abnormal load upon normal bone (the fatigue fracture) and relatively normal loading upon abnormal bone (the insufficiency fracture).^(^ ^2^ ^)^ The former has been suggested to occur particularly commonly in the physically active individuals including but not limited to track and field athletes, long distance runners, dancers, and military recruits.^(^ ^3^, ^4^ ^)^ Compared with men, active women (eg, female athletes and military) tend to have a higher incidence of fatigue fractures.^(^ ^5^, ^6^ ^)^ Anatomically, the tibia, tarsal bones, and metatarsals are the most frequently affected sites.^(^ ^7^, ^8^ ^)^ On the other hand, elderly and postmenopausal women have been reported most at risk for developing insufficiency fractures,^(^ ^9^ ^)^ which typically involve the spine, sacrum, and pelvis^(^ ^10^, ^11^ ^)^ and can be found in conditions of vitamin deficiency,^(^ ^12^ ^)^ osteomyelitis, hypophosphatasia,^(^ ^13^ ^)^ and fractures associated with long‐term bisphosphonate use.^(^ ^14^ ^)^

Different from elderly patients with a low‐energy insufficiency fracture, fatigue fractures are generally uncomplicated and managed conservatively with protected or limited weight‐bearing and physical therapy.^(^ ^15^ ^)^ Patients could return gradually to sport‐specific activity when they are symptom‐free.^(^ ^16^ ^)^ However, fatigue fractures found in sites with challenging blood supply and maximal tensile load (eg, lateral femoral neck, anterior tibia, proximal second and fifth metatarsal) are at high risk due to the predilection of progression to complete fracture, delayed union, or nonunion.^(^ ^17^, ^18^ ^)^ High‐risk fatigue fractures respond poorly to conservative treatment and may cause significant morbidity.^(^ ^19^ ^)^ Thus, more aggressive evaluation and treatment (immediate no weight‐bearing cast immobilization, surgical fixation, etc.) are required when managing these challenging stress fractures, which should be similar to dealing with acute fractures.^(^ ^20^, ^21^, ^22^ ^)^

The etiology of stress fractures is multifactorial.^(^ ^23^ ^)^ As to fatigue fracture, the most common risk factor is abrupt increase in activity or training patterns.^(^ ^24^ ^)^ A longitudinal study of 5000 Finnish male military recruits demonstrated that a higher level of high‐intensity activity before entering training helped to protect against a stress fracture.^(^ ^25^ ^)^ Bearing a load of sufficient magnitude, bone deforms through its elastic range, out of which the bone deformation is not adequate to absorb the strain, leading to microscopic cracking, diffuse damage of bone tissue, and persistent plastic deformity.^(^ ^26^, ^27^ ^)^ Accumulation of microcrack with continued loading will be translated ultimately into a discontinuity within the cortical bone that is a stress fracture.^(^ ^28^ ^)^ It has been theorized that osteocyte apoptosis, bone marrow edema, localized contractile muscle pressure, and vitamin D (25(OH)D) deficiency may have a role in above‐mentioned pathogenetic processes.^(^ ^29^, ^30^, ^31^, ^32^, ^33^ ^)^ Of note, the process of bone remodeling in response to increased mechanical loading is of double effect. Namely, it may aid to repair microcracks while also temporarily decreasing bone elastic modulus.^(^ ^34^, ^35^ ^)^ Only limited histomorphometric data are available and the link between stress fracture risk and bone remodeling–induced increase in porosity remains to be demonstrated experimentally.^(^ ^36^ ^)^ In addition, few studies investigate the bone components to elucidate the value of biomolecular content in the fatigue fracture. It is therefore of clinical importance to determine how the biochemical information of bone in patients with fatigue fractures relate to its histological parameters in order to gain a deeper understanding of related physiological processes.

We report on a middle‐aged woman, otherwise healthy, but with multiple fatigue fractures with delayed healing. Iliac crest bone histomorphometry revealed uncoupled bone remodeling with low bone formation, marked peritrabecular fibrosis, and significantly increased bone resorption. The biochemical profile of her bone tissue was established through Fourier transform infrared spectroscopic imaging (FTIRI) technique.

## 2 Patient and Methods

### 2.1 Medical history

**Fig. 1:**
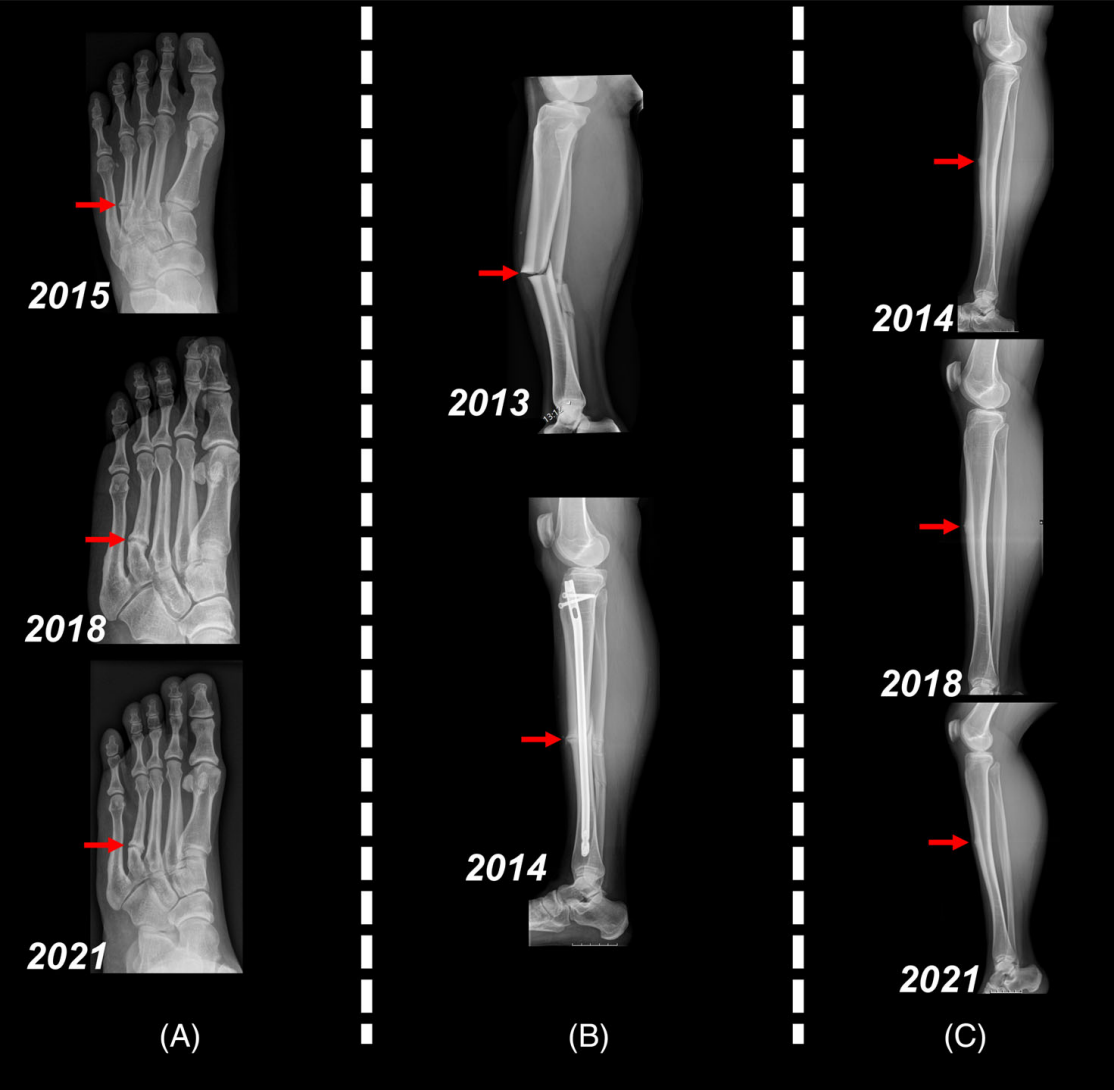
Radiographs of the left proximal fourth metatarsal (*A*), right tibial diaphysis and fibula (*B*), and left tibial diaphysis (*C*) showing fatigue fractures and delayed healing (indicated in red).

This 35‐year‐old White woman was referred by a local endocrinologist for unexplained fractures in 2014. Her medical history was significant during the years 2008 to 2014 with four fractures. The first two were metatarsal stress fractures in 2008 and 2012 with remarkably slow fracture healing. The third was tibial diaphysis dislocated fracture on the right side due to slip fall when walking in 2013. A couple of months later in 2014, the fourth fracture involved her left tibia without trauma. Both‐side tibial fractures have suggested to be the typical fatigue ones showing horizontal fracture line and cortical thickening (Fig. 1).

The patient weighed 66.7 kg and her height was 162 cm. Her bone mineral density (BMD) in spine and hip were within the high‐normal reference (*T*‐score +1.0 and +0.5, respectively). She was otherwise healthy, and her clinical status was normal. There was no history of smoking or excessive alcohol use. The skin was clean and there were no bruises or stretch marks. There were no signs for the secretion of excessive cortisol. The scleras were normal. The auscultation of heart and lungs was neat, and her squat was effortless. She used calcium‐vitamin D supplements and had contraceptive pills as the only medication for 21 years (MELIANE 1997–2015, QLAIRA 2016–2018). She was rather physically active in sports. Before fractures, she had been jogging 5 to 6 kilometers (km) 3 to 4 times/week. After the tibial fracture, her exercise included gym, biking, and aqua jogging (slowly running in a pool).

In 2018, the patient was followed up and her clinical situation was found unchanged. Daily normal movements caused no symptoms; however, the patient reported similar pain in her front legs and left foot after several days of exertion. She has been managing performing light exercises such as long‐distance walking (9 km). However, she was not able to run. X‐ray examination revealed cortical thickening in the left front tibia, and the fracture line could no longer be distinguished. In the proximal fourth metatarsal (MT4), old stress fracture was still visible, whereas there was a clear blurring compared with the X‐ray images in 2015 (Fig. 1). Because of slowly improved fatigue fractures in leg and foot, no new fractures, and no diagnosis found for bone situation, the patient was continually followed up in 3 years.

### 2.2 Biochemical findings

Biochemical evaluation was applied at the time of her left tibial fracture (May 2014) upon referral to us. Hormonal examination demonstrated normal thyroid and parathyroid function (TSH 2.01 mU/L; PTH 21 ng/L) as well as normal prolactin (313 mU/L) and NTx (24 nmoL/mmoL creatinine). In dexamethasone suppression test (1 mg test), the plasma cortisol response was normal (1333–99 nmol/L). Long‐term ingestion of contraceptive pills explained slightly high baseline cortisol level, as well as resulted in low follicle‐stimulating hormone (FSH; 0.8 U/L) and low estradiol (<0.04 nmol/L) levels. Serum vitamin D balance was normal: 25(OH)D was 90 nmol/L, 1,25(OH)_2_D was 189 pmol/L. Celiac antibody (tTg‐IgA) was normal (0.2 U/mL) and IgA was normal (1.06 g/L). Plasma calcium was normal (2.18 mmol/L), whereas ionized calcium was a bit low (1.15 mmol/L). Electrolytes, creatinine, and blood cell count were normal (Table 1).

**Table 1:** Patient's Biochemistry

Biochemical parameters	May 5, 2014	June 5, 2014	Reference range
Serum			
Parathyroid hormone (PTH)	21		15–65 ng/L
25(OH) vitamin D	90		>40 nmol/L
1,25(OH)_2_ vitamin D	189		63–228 pmol/L
Tissue transglutaminase IgA (tTg‐IgA)	0.2		<7 U/mL
Calcium	1.15		1.16–1.3 mmol/L
PH	7.43		7.35–7.45
Estradiol	<0.04		0.18–2.36 nmol/L
Plasma			
Thyroid stimulating hormone (TSH)	2.01		0.5–5.0 mU/L
Alkaline phosphatase	53		35–105 U/L
Phosphorus	1.07		0.76–1.41 mmol/L
Immunoglobulin A (IgA)	1.06		0.52–4.02 g/L
Albumin	40		36–48 g/L
Creatinine	72		50–90 umol/L
Natrium	137		137–144 mmol/L
Kalium	3.4		3.4–4.7 mmol/L
Prolactin	313		102–496 mU/L
Calcium	2.18		2.15–2.51 mmol/L
Follicle‐stimulating hormone (FSH)	0.8		<7.9 U/L
Cortisol	1333	99	nmol/L
Urine			
24 hr calcium (volume = 2.3 L)	3.95		1.25–7.5 mmol/L
N‐telopeptide of type I collagen (NTx)		24	<65 nmoL/mmoL
Creatinine		4.4	1.7–19.4 mmol/L
Complete blood count (CBC)			
White blood cells (WBC)	5.8		3.4–8.2 × 10^**9**^/L
Red blood cells (RBC)	4.14		3.9–5.2 × 10^**12**^/L
Hemoglobin (HGB)	122		117–155 g/L
Hematocrit (HCT)	0.38		0.35–0.46 L/L
Mean corpuscular volume (MCV)	91		82–98 fL
Mean corpuscular hemoglobin (MCH)	30		27–33 pg
Mean corpuscular hemoglobin concentration (MCHC)	325		315–360 g/L
Platelet count (PLT)	281		150–360 × 10^**9**^/L

### 2.3 Mutation analysis

A genetic analysis has failed to find a mutation in the Janus kinase 2 gene (JAK2 V617F mutation) known to be classically associated with chronic myeloproliferative diseases (eg, myeloproliferative neoplasms [MPN]). We did not make any other genetic screening. For instance, no clinical signs related to loss‐of‐function mutations in Wnt pathway–related genes were detected.

### 2.4 Bone biopsy

Transiliac bone biopsy after tetracycline labeling was obtained from the patient in May 2014. A total of 16 cadavers with no history of medical conditions or use of drugs known to affect their bone metabolism served as healthy controls. They were divided into two subgroups: histomorphometric analysis control (*n* = 6) and FTIRI analysis control (*n* = 11). In addition, we used the histomorphometric data reported by Recker and colleagues in 2018^(^ ^37^ ^)^ as the other source for normal range of each parameter. Ethical approval for collection of samples was granted by the National Authority for Medicolegal Affairs (permission number 5783/04/044/07).

Samples were dehydrated in ethanol (70%) for at least 48 hours and embedded in polymethylmethacrylate (PMMA) according to standard protocols.^(^ ^38^ ^)^ After embedding, 5‐μm‐thick sections were cut using a microtome (Reichert‐Jung; Cambridge Instruments, Heidelberg, Germany) for histomorphometric analysis before staining with modified Masson Goldner trichrome stain. Three‐μm‐thick unstained sections were cut and placed on ZnSe windows for FTIRI analysis.

#### 2.4.1 Histological analysis

Quantitative bone histomorphometry was performed using Osteomeasure system (OsteoMetrics, Atlanta, GA, USA). The nomenclature, abbreviations, and parameters follow the recommendations by the American Society for Bone and Mineral Research (ASBMR).^(^ ^39^ ^)^ Each sample was evaluated in turn under bright light, polarization, and fluorescence microscopy using a magnification of ×200 (Fig. S1). Measurements covered the complete cancellous bone area, and parameters representing different types of bone structure were computed accordingly (Table S1).

#### 2.4.2 Fourier transform infrared spectroscopic analysis

The compositional analysis was carried out using the FTIR‐MS system (Agilent Cary 670/620; Agilent Technologies, Santa Clara, CA, USA) equipped with a focal plane array (FPA) detector (pixel size: 128 × 128, field of view [FOV] 140 × 140 μm^2^) and an optical microscope. The spatial pixel size was 5.5 × 5.5 μm^2^ and the spectral resolution was set to 4 cm^–1^. The number of scans per pixel was set to 100 and averaged for an improved signal‐to‐noise ratio. A background scan was first acquired from a clear window and corrected for each measured spectrum. Then the FTIR‐MS system was used to collect (averaged) infrared spectra in a point‐by‐point manner from both cancellous bone and cortical bone (Fig. S2). Parameters reflecting the spatial biochemical composition of bone tissues were generated. The spectra were collected in a wavenumber range between 3800 cm^–1^ and 750 cm^–1(^ ^40^ ^)^ (Table S1).

## 3 Results

### 3.1 Histomorphometric findings

**Fig. 2:**
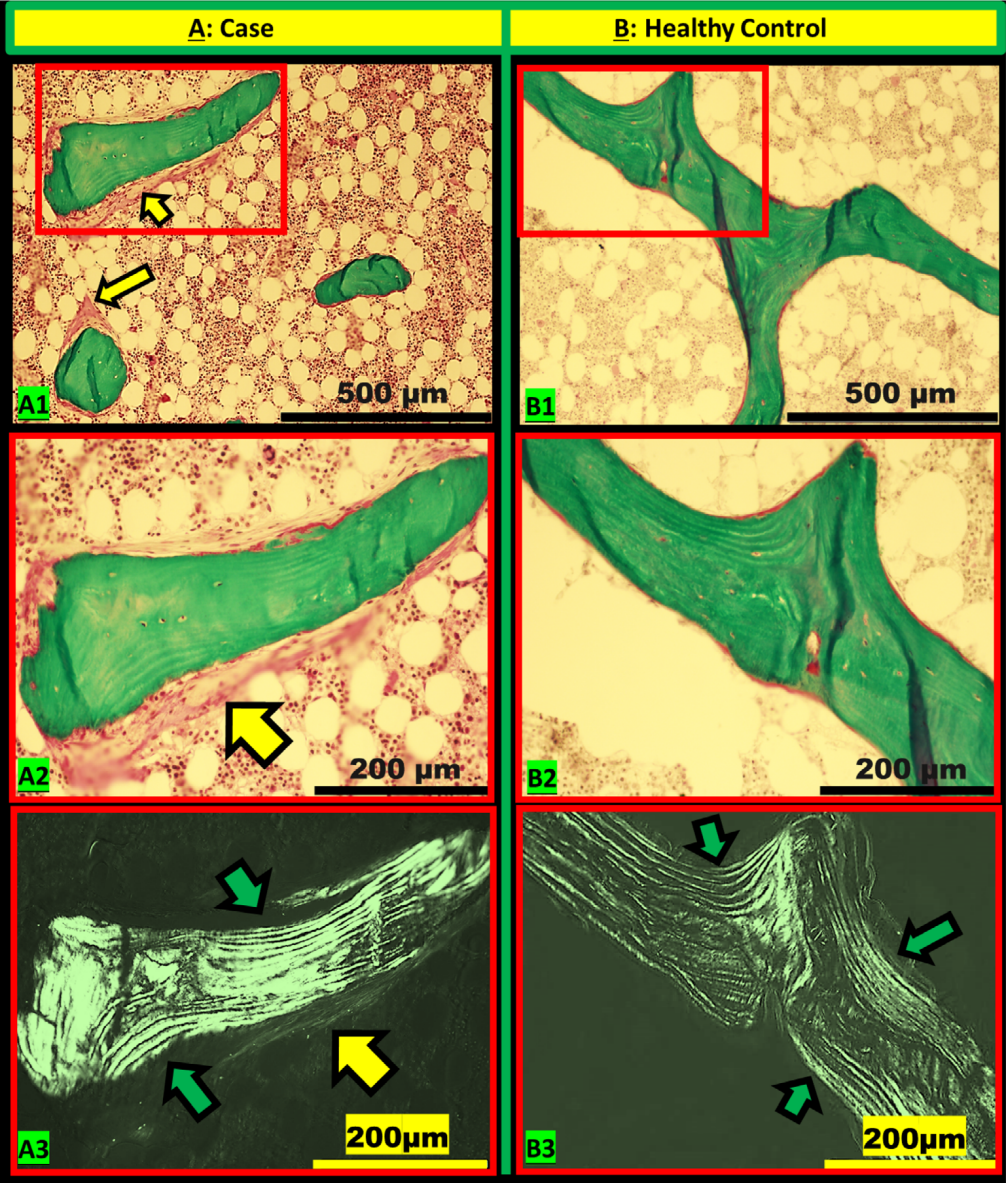
Typical microarchitectures of cancellous bone of iliac crest specimens under light microscopy (*A1‐2*; *B1‐2*) and polarization microscopy (*A3*; *B3*) are exemplified by our case (female, age 35 years) and one healthy control (female, age 38 years). The magnified images (highlighted by red rectangle) demonstrate the peritrabecular fibrosis (indicated by yellow arrow) and parallel‐organized osseous lamellae (indicated by green arrow). Masson Goldner trichrome stain, magnification 100× (*A1*; *B1*); 200× (*A2‐3*; *B2‐3*).

In the analyses of static parameters, osteoid volume (OV/BV), osteoid surface (OS/BS), wall thickness (W.Th), trabecular number (Tb.N) were found within normal ranges of both control materials. Bone volume (BV/TV), osteoblast surface (Ob.S/BS), trabecular thickness (Tb.Th), trabecular separation (Tb.Sp) had mild decline compared to the published data, and osteoid thickness (O.Th) had mild elevation compared to our normal control. Since these values were slightly different from the lower/upper limit of the normal range, also because the assay normal ranges varied between measurements, we consider above‐mentioned parameters as normal. Moreover, parameters describing marrow fibrosis were markedly elevated compared to our controls: fibrosis volume (Fb.V/TV) >14x, fibrosis interface (Fb.I/BS) >5x, fibrosis thickness (Fb.Th) >1.9x the upper limit of the normal range (Figure 2). Parameters related to bone resorption showed remarkable elevation compared to both Recker’s data and our controls: eroded surface (ES/BS) >6x and >3.9x, osteoclast surface (Oc.S/BS) >20x and >6x the upper limit of the normal range, respectively. In our case, no tetracycline double labels were seen in cancellous bone and only a short one was seen in the endo‐cortex. Correspondingly, compared to Recker’s normal range, we found low‐normal bone formation rate/bone surface referent (BFR/BS); declined mineralizing surface at both bone surface and osteoid referents (MS/BS, MS/OS) and declined activation frequency (Ac.f); increased mineralization lag time (Mlt); mineral apposition rate (MAR) and remodeling cycle duration related parameters: formation period (FP), resorption period (Rs.P), remodeling period (Rm.P) (Table 2).

**Table 2:** Quantitative Trabecular Bone Histomorphometry of Iliac Crest Specimens from the Case, Published Normal Data,^(^ ^37^ ^)^ and Our Normal Controls

Parameters	Case	Published normal data for premenopausal women		Normal controls (*n* = 6)	
		Normal mean (SD)	Normal ranges	Normal mean (SD)	Normal ranges
Static					
BV/TV (%)	15.04	22.02 (5.55)	16.47–27.57	15.3 (5.6)	10.17–20.43
OV/BV (%)	1.45	0.98 (0.64)	0.34–1.62	1.35 (0.54)	0.86–1.84
Fb.V/TV (%)	1.42	—	—	0.0625 (0.036)	0.029–0.096
OS/BS (%)	13.07	10.61 (6.51)	4.1–17.12	16.0 (5.44)	11.01–20.94
ES/BS (%)	20.40	2.11 (1.23)	0.88–3.34	4.3 (0.91)	3.48–5.13
Fb.I/BS (%)	22.60	—	—	2.56 (1.44)	1.25–3.88
Ob.S/BS (%)	0.66	3.67 (2.67)	1.0–6.34	1.11 (1.18)	0.03–2.19
Oc.S/BS (%)	5.89	0.11 (0.16)	−0.05–0.27	0.55 (0.44)	0.14–0.95
Tb.Th (μm)	88.0	154 (33)	121–187	92.5 (20.6)	73.64–111.31
O.Th (μm)	4.84	6.0 (2.2)	3.8–8.2	3.72 (0.56)	3.21–4.23
Fb.Th (μm)	18.94	—	—	7.6 (2.3)	5.55–9.66
W.Th (μm)	41.79	39.4 (7.6)	31.8–47	42.1 (4.76)	37.8–46.5
Tb.Sp (μm)	497.4	696 (104)	592–800	569.4 (220.1)	368.4–770.3
Tb.N (#/mm)	1.71	1.43 (0.19)	0.7–6.15	1.61 (0.39)	1.25–1.96
Dynamic					
BFR/BS (μm^**3**^/μm^**2**^/yr)	1.07	7.16 (6.4)	0.75–13.56	—	—
MS/BS (%)	0.23	3.4 (2.7)	0.7–6.1	—	—
MS/OS (%)	1.74	35.6 (25.9)	9.7–61.5	—	—
MAR (μm/d)	1.29	0.45 (0.16)	0.29–0.61	—	—
Mlt (d)	216.2	56.1 (43.4)	12.7–99.5	—	—
Omt (d)	3.8	14.2 (6.0)	8.2–20.2	—	—
FP (yr)	5.12	1.12 (1.15)	−0.03–2.27	—	—
Rs.P (yr)	2.31	0.23 (0.31)	−0.08–0.54	—	—
Rm.P (yr)	13.10	1.35 (1.29)	0.06–2.64	—	—
Ac.f (#/yr)	0.03	0.15 (0.11)	0.04–0.26	—	—

Abbreviations: Ac.f, activation frequency; BFR/BS, bone formation rate (bone surface referent); BV/TV, bone volume; ES/BS, eroded surface; Fb.I/BS, fibrosis interface; Fb.Th, fibrosis thickness; Fb.V/TV, fibrosis volume; FP, formation period;MAR, mineral apposition rate; Mlt, mineralization lag time; MS/BS, mineralizing surface (bone surface referent); MS/OS, mineralizing surface (osteoid referent); O.Th, osteoid thickness; Ob.S/BS, osteoblast surface; Oc.S/BS, osteoclast surface; Omt, osteoid maturation time; OS/BS, osteoid surface; OV/BV, osteoid volume; Rm.P, remodeling period; Rs.P, resorption period; Tb.N, trabecular number; Tb.Sp, trabecular separation; Tb.Th, trabecular thickness; W.Th, wall thickness.

### 3.2 FTIRI findings

Compared with our normal controls, both cancellous bone and cortex in this case demonstrated remarkably decreased carbonate‐related parameters: carbonate‐to‐matrix ratio (C/M), carbonate‐to‐phosphate ratio (C/P), etc. Collagen cross‐linking ratio (XLR) and crystallinity had been found high‐normal in cancellous bone and increased in cortical bone. Mineral‐to‐matrix ratio (M/M) had a decline in cancellous bone, and acid phosphate substitution (APS) was within the normal range in both bone tissues (Table 3).

**Table 3:** Compositional Quantification (FTIRI) of Trabecular Bone and Cortical Bone of Iliac Crest Specimens From the Case and Our Normal Controls

Parameters	Case	Normal controls (*n* = 11)	
		Normal mean (SD)	Normal ranges
Trabecular bone			
Cross‐linking ratio (XLR)	2.73	2.50 (0.37)	2.13–2.87
Crystallinity	1.09	1.07 (0.03)	1.04–1.1
Acid phosphate substitution (APS)	1.33	1.48 (0.24)	1.24–1.72
Mineral to matrixphosphate: amide I	3.84	4.71 (0.76)	3.95–5.47
Carbonate: matrix	5.60	13.1 (3.4)	9.7–16.5
Carbonate: phosphate	1.78	2.7 (0.45)	2.25–3.15
Cortical bone			
XLR	2.86	2.5 (0.22)	2.28–2.72
Crystallinity	1.19	1.14 (0.04)	1.1–1.18
APS	1.49	1.52 (0.14)	1.38–1.66
Mineral to matrix phosphate: amide I	4.60	4.8 (0.46)	4.34–5.26
Carbonate: matrix	5.28	9.6 (1.51)	8.09–11.11
Carbonate: phosphate	1.15	2.05 (0.33)	1.72–2.38

## 4 Discussion

Based on iliac crest biopsy, the present study investigated histomorphometric and biochemical characteristics of the patient suffering multiple fatigue fractures. Because of the likelihood of progression to outright fractures and limited amount in collection,^(^ ^41^, ^42^ ^)^ we avoided taking specimens from the nonunion site. The advantage of our approach is its ability to represent general bone remodeling. Local structural change of the fracture site may mimic, for example, that of the aggressive bone tumor caused by osteoblastic reparative callus, leading to histological confusion.^(^ ^43^ ^)^

Bone remodeling aiming to repair fatigue damages is in a non‐random, lesion‐specific manner, which has been termed “targeted remodeling.”^(^ ^44^, ^45^ ^)^ In specific, with increased loading, microcrack generation and its development in turn lead to osteocyte apoptosis, subsequent expression of cytokines related to the recruitment of osteoclast precursors, and removal of microarchitecturally damaged bone.^(^ ^46^, ^47^ ^)^ Although both bone formation and bone resorption increase with loading activities,^(^ ^48^ ^)^ repetitive strains could stimulate osteoclasts to resorption at a faster rate than osteoblasts can form new bone.^(^ ^49^ ^)^ In our case, eroded surface and osteoclast surface in iliac crest were found as a multiple of or even dozens of the upper limit of the normal range, suggesting explicitly high bone turnover. However, relatively low‐normal osteoblast surface and bone formation rate, in conjunction with a trend toward prolongation of remodeling duration, suggested inactive bone formation. Our patient had been using combined oral contraceptives (COC) more than 20 years. It is unclear if this distinct uncoupling between osteoclastic and osteoblastic activities has anything to do with the long‐term use of COC. Allali and colleagues have reported the finding of a decrease in bone turnover, based on biochemical markers, in premenopausal women who used COC for years.^(^ ^50^ ^)^ We found one short double label of tetracycline in the endo‐cortex, despite which the marginally elevated thickness of unmineralized osteoid along with low bone formation may indicate abnormal bone mineralization.^(^ ^51^ ^)^ The uncoupling in her bone remodeling could lead to bone formation lags behind bone resorption, resulting in temporary dominance of osteoclastic recruitment. The resultant porous spaces represent focal negative bone balance, potentially introducing an acute increase in porosity that decreases bone stiffness until fatigue fracture occurring.^(^ ^52^ ^)^

It is noteworthy that we found striking presence of fibrous connective tissue, which was of proximity to cancellous bone (peritrabecular fibrosis). Related amount was much higher than the upper limit of the normal range in levels of volume, surface, and width. The peritrabecular fibrosis has been regarded as the key indicator of high‐turnover bone diseases (eg, osteitis fibrosa) when fibroblast‐like cells produce much irregularly organized extracellular matrix in response to persistently increased PTH levels.^(^ ^53^ ^)^ Bone pathologies mediated by hyperparathyroidism and elevated turnover are characterized by active remodeling in both bone formation and resorption.^(^ ^54^ ^)^ Given the fact that our patient had normal functioning parathyroid and her osteoblastic osteogenesis was low, the histological manifestation in our case seems to be irrelevant. Thus, increased fibrous tissue shown in her bone marrow argues that peritrabecular fibrosis might be independent of prolonged and increased exposure to PTH. Abundant fibrous connective tissue represents increased function of fibroblast‐like cells, which can have precursors of hematologic origin,^(^ ^55^ ^)^ or from mesenchymal stem cells^(^ ^56^ ^)^ or from osteoprogenitor cells (preosteoblasts).^(^ ^57^ ^)^ With normal PTH levels, preosteoblasts are supposed to differentiate to osteoblastic progenitors and further into osteoblasts.^(^ ^58^ ^)^ Although the exact pathogenetic processes of abundant peritrabecular fibrosis in our patient are yet unknown, there is a possibility that increased fibrous tissue may indicate decreased differentiation of preosteoblast‐origin fibroblasts into osteoblasts and consequently decreased bone formation.

On the other hand, agreeing with our histomorphometric findings, the FTIRI analyses also demonstrate a decrement in her bone mineralization: decline in both M/M and C/M. They provide similar information about the bone mineral content based on phosphate and carbonate, respectively. Beside initial deposition of minerals, process of mineralization also comprises their maturation and perfection in number, size, and quality.^(^ ^59^ ^)^ In this regard, carbonate substitution calculated as C/P should be considered together with M/M and C/M as reliable indicators of bone mineralization.^(^ ^60^ ^)^ Our finding that lower C/M, M/M, and C/P compared with the normal controls is concordant with previous FTIRI investigations of osteoporotic cases,^(^ ^61^ ^)^ femoral neck fractured cases,^(^ ^62^ ^)^ and fatigue fractured cases,^(^ ^63^ ^)^ demonstrating that abnormal bone mineralization, could correlate with poor mechanical strength, low toughness, microcrack generation, and fracture.^(^ ^64^, ^65^ ^)^ Moreover, the crystallinity and collagen cross‐linking ratio (XLR) were found higher in our patient. The former clarifies the mineral crystal size and perfection, whereas the latter describes the collagen maturity showing the developmental stage of the collagen network.^(^ ^66^, ^67^, ^68^ ^)^ Both have been found to increase during maturation and with aging.^(^ ^69^ ^)^ In addition, crystallinity has been reported increased in fractures,^62^, ^70^ ^)^ and elevated collagen maturity was suggested to contribute to bone weakening and increased fracture risk.^(^ ^71^ ^)^ These findings are in line with our results. Nevertheless, Mata‐Miranda and colleagues have found that XLR was lower in their fatigue fracture group than in the health group.^(^ ^63^ ^)^ It should be noted that most of their study subjects with fatigue fracture were younger than 30 years, who have not reached their higher peak bone mass, matrix maturity, and strength.

Our patient had several typical risk factors of fatigue fracture. First, women are more likely to suffer fatigue fractures than men, especially who participate in intense sports activity frequently.^(^ ^72^, ^73^ ^)^ She has been quite active in sports, although she is not an athlete and has no strenuous training volume. Second, female adherence to oral contraceptive pills (OCP) could have menstrual disturbance and being physically active may accelerate related dysfunction.^(^ ^74^, ^75^, ^76^ ^)^ It was shown that the risk of subsequent fractures among women who had ever used OCPs was significantly higher than those who had not.^(^ ^77^ ^)^ Female athletes with menstrual disturbances were found two to four times more likely to suffer from a fatigue fracture than their eumenorrheic teammates.^(^ ^78^ ^)^ Third, a past history of previous stress fracture may work as an intrinsic risk factor for coming ones.^(^ ^79^, ^80^ ^)^

Up to present, she has not developed new fractures and regular movement causes no symptoms. Discomfort shows up in foot and tibial shaft after exertion (eg, running), where old fatigue fractures have been healing gradually with fracture lines getting blurred. She gave birth to a healthy child in 2020.

Although fatigue‐related stress fracture is widely encountered in sports medicine and rheumatology, the underlying pathophysiology has yet to be completely defined. In this report, we explored the histological etiology of lag osteogenesis in bone remodeling as well as showed bone biochemical differences between the healthy bone and patient with multiple fatigue fractures. According to our findings, we conclude that peritrabecular fibrosis could play a role in the decreased osteogenesis and delayed fracture healing, and fatigue fractures may relate to the increased collagen maturity and decreased mineralization in bone tissue.

## Conflict of Interest

All authors have declared that no conflicts of interest exist.

5

### Peer Review

The peer review history for this article is available at https://publons.com/publon/10.1002/jbm4.10598.

## Supporting information


Fig. S1.

Fig. S2.

**Table S1.** Quantitative Parameters Utilized in Histomorphometric and FTIRI Analyses
