# Supplementary figures and images for: Generalized Uncoupled Bone Remodeling Associated With Delayed Healing of Fatigue Fractures
Source: JBMR Plus. 2022 Jan 19;6(3):e10598. doi: 10.1002/jbm4.10598 (PMC8914151; doi:10.1002/jbm4.10598)

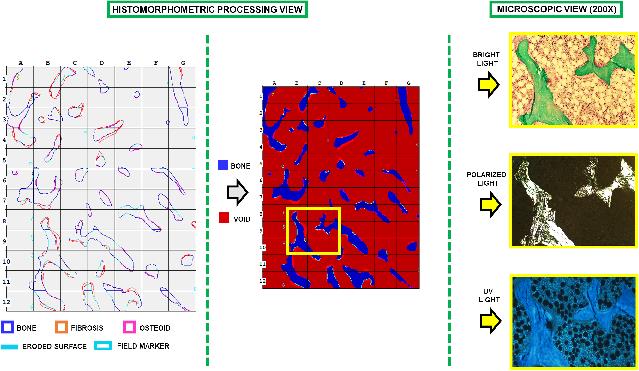

Supplement: Supplementary file 1 — Fig. S1. [file JBM4-6-e10598-s001.jpg]

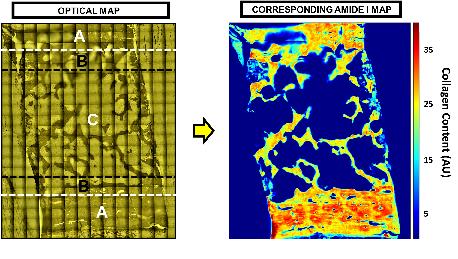

Supplement: Supplementary file 2 — Fig. S2. [file JBM4-6-e10598-s002.jpg]
