# Supplementary material for: Generalized Uncoupled Bone Remodeling Associated With Delayed Healing of Fatigue Fractures
Source: JBMR Plus. 2022 Jan 19;6(3):e10598. doi: 10.1002/jbm4.10598 (PMC8914151; doi:10.1002/jbm4.10598)
Supplement: Supplementary file 3 — Table S1. Quantitative Parameters Utilized in Histomorphometric and FTIRI Analyses [file JBM4-6-e10598-s003.docx]

| **Appendix**. Quantitative parameters utilized in histomorphometric and FTIRI analyses. | |  |
| --- | --- | --- |
|  |  |  |
| Parameters |  |  |
|  |  |  |
| ***Trabecular bone histomorphometry*** | |  |
| *KEY parameters of trabecular microstructure* | *Abbreviation (unit)* |  |
| Bone volume | BV/TV (%) |  |
| Osteoid volume | OV/BV (%) |  |
| Fibrosis volume | Fb.V/TV (%) |  |
| Osteoid surface | OS/BS (%) |  |
| Eroded surface | ES/BS (%) |  |
| Fibrosis interface | Fb.I/BS (%) |  |
| Osteoblast surface | Ob.S/BS (%) |  |
| Osteoclast surface | Oc.S/BS (%) |  |
| Trabecular thickness | Tb.Th (μm) |  |
| Osteoid thickness | O.Th (μm) |  |
| Fibrosis thickness | Fb.Th (μm) |  |
| Wall thickness | W.Th (μm) |  |
| Trabecular separation | Tb.Sp (μm) |  |
| Trabecular number | Tb.N (#/mm) |  |
| Bone formation rate (bone surface referent) | BFR/BS (μm**^3^**/μm**^2^**/yr) |  |
| Mineralizing surface (bone surface referent) | MS/BS (%) |  |
| Mineralizing surface (osteoid referent) | MS/OS (%) |  |
| Mineral apposition rate | MAR (μm/d) |  |
| Mineralization lag time | Mlt (d) |  |
| Osteoid maturation time | Omt (d) |  |
| Formation period | FP (yr) |  |
| Resorption period | Rs.P (yr) |  |
| Remodeling period | Rm.P (yr) |  |
| Activation frequency | Ac.f (#/yr) |  |
| ***FTIRI*** | |  |
| *KEY parameters of bone composition* | *Wavenumbers [cm****^−1^****]* |  |
| Collagen cross-linking ratio | 1660:1690 |  |
| Crystallinity | 1030:1020 |  |
| Acid phosphate substitution | 1127:1096 |  |
| Mineral/matrix (Phosphate/amide I) (%) | (1200-900):(1720-1585) |  |
| Carbonate/matrix (Carbonate/amide I) (%) | (890-850):(1720-1585) |  |
| Carbonate/phosphate (%) | (890-850):(1200-900) |  |
